# Deep Learning Automation of Kidney, Liver, and Spleen Segmentation for Organ Volume Measurements in Autosomal Dominant Polycystic Kidney Disease

**DOI:** 10.3390/tomography8040152

**Published:** 2022-07-13

**Authors:** Arman Sharbatdaran, Dominick Romano, Kurt Teichman, Hreedi Dev, Syed I. Raza, Akshay Goel, Mina C. Moghadam, Jon D. Blumenfeld, James M. Chevalier, Daniil Shimonov, George Shih, Yi Wang, Martin R. Prince

**Affiliations:** 1 Department of Radiology, Weill Cornell Medicine, Cornell University, New York, NY 10065, USA; ars4017@med.cornell.edu (A.S.); djr4003@med.cornell.edu (D.R.); kut2002@med.cornell.edu (K.T.); hrd2001@med.cornell.edu (H.D.); sir4001@med.cornell.edu (S.I.R.); akshay.k.goel@gmail.com (A.G.); mch4003@med.cornell.edu (M.C.M.); ges9006@med.cornell.edu (G.S.); 2 The Rogosin Institute and Department of Medicine Weill Cornell Medicine, Cornell University, New York, NY 10065, USA; jdblume@nyp.org (J.D.B.); jac9014@nyp.org (J.M.C.); das2041@nyp.org (D.S.); 3 Departments of Radiology at Weill Cornell Medicine and Biomedical Engineering, Cornell University, New York, NY 10065, USA; yiwang@med.cornell.edu; 4 Columbia College of Physicians and Surgeons, Cornell University, New York, NY 10027, USA

**Keywords:** liver volume, kidney volume, spleen volume, ADPKD, artificial intelligence, interobserver variability, machine learning

## Abstract

Organ volume measurements are a key metric for managing ADPKD (the most common inherited renal disease). However, measuring organ volumes is tedious and involves manually contouring organ outlines on multiple cross-sectional MRI or CT images. The automation of kidney contouring using deep learning has been proposed, as it has small errors compared to manual contouring. Here, a deployed open-source deep learning ADPKD kidney segmentation pipeline is extended to also measure liver and spleen volumes, which are also important. This 2D U-net deep learning approach was developed with radiologist labeled T2-weighted images from 215 ADPKD subjects (70% training = 151, 30% validation = 64). Additional ADPKD subjects were utilized for prospective (n = 30) and external (n = 30) validations for a total of 275 subjects. Image cropping previously optimized for kidneys was included in training but removed for the validation and inference to accommodate the liver which is closer to the image border. An effective algorithm was developed to adjudicate overlap voxels that are labeled as more than one organ. Left kidney, right kidney, liver and spleen labels had average errors of 3%, 7%, 3%, and 1%, respectively, on external validation and 5%, 6%, 5%, and 1% on prospective validation. Dice scores also showed that the deep learning model was close to the radiologist contouring, measuring 0.98, 0.96, 0.97 and 0.96 on external validation and 0.96, 0.96, 0.96 and 0.95 on prospective validation for left kidney, right kidney, liver and spleen, respectively. The time required for manual correction of deep learning segmentation errors was only 19:17 min compared to 33:04 min for manual segmentations, a 42% time saving (*p* = 0.004). Standard deviation of model assisted segmentations was reduced to 7, 5, 11, 5 mL for right kidney, left kidney, liver and spleen respectively from 14, 10, 55 and 14 mL for manual segmentations. Thus, deep learning reduces the radiologist time required to perform multiorgan segmentations in ADPKD and reduces measurement variability.

## 1. Introduction

Autosomal dominant polycystic kidney disease (ADPKD) is the most common inherited cause of chronic kidney disease (CKD), affecting over 10 million people worldwide [1]. The ADPKD phenotype is characterized by the enlargement of kidney cysts, tubulointerstitial fibrosis, and progression to end stage kidney disease (ESKD). Extrarenal manifestations include cystic involvement of the liver, pancreas [2], prostate [3] and arachnoid [4], as well as seminal megavesicles [5], splenomegaly [6] and cardiovascular effects including pericardial effusions [7], aortic ectasia, dolichoectasia and saccular intracranial aneurysms [8]. In particular, the liver can be enlarged by cysts compressing hepatic veins and bile ducts, requiring cyst fenestration, partial hepatic resection or liver transplantation [9]. Splenomegaly, without associated spleen cysts, is a common feature. Although its cause and clinical significance are not established, it can contribute to mass effects in the left upper abdomen, potentially causing early satiety and the challenge of differentiating among other causes of splenomegaly [6].

Standard biomarkers for renal function, such as creatinine-based estimates of the glomerular filtration rate, are imprecise for tracking ADPKD progression because glomerular hyperfiltration and other compensatory mechanisms obscure the loss of functioning renal mass [10,11,12,13,14,15]. Similarly, routine blood tests for liver and spleen function do not correlate with ADPKD changes in those organs.

Several large studies have demonstrated that total kidney volume (TKV), indexed to patient height (ht-TKV), identifies ADPKD patients at highest risk for progression to ESKD [10]. In clinical trials of tolvaptan, the only drug approved for the treatment of ADPKD, the rate of increase in TKV was attenuated in patients treated with tolvaptan compared with control groups. Accordingly, MRI TKV has become an important ADPKD biomarker that is now considered the standard for evaluating ADPKD [16].

Total kidney volume (TKV) can be approximated as an ellipsoidal solid with kidney length, width and depth measurements, with stereology [17] or measured more accurately with laborious manual contouring of kidney outlines on every slice of CT or MRI scans [18,19,20,21]. Recently, deep learning-based methods have semi-automated TKV measurement [18,19,20,21] by producing an initial prediction of renal contours on CT or MRI that can be rapidly refined by an expert observer. This eliminates the need to manually draw every contour of the cystic kidneys, [22] thereby increasing the efficiency of accurate TKV measurement. Table 1 summarizes the existing literature for deep learning-based organ volume measurements in ADPKD using CT [23,24,25,26,27], ultrasound [28] and MRI [22,29,30,31,32,33,34,35]. MRI has the advantage over CT of not requiring ionizing radiation, which is particularly important, for these organ volume measurements are repeated many times over the patient’s lifetime, and MRI has higher resolution compared to ultrasound. Only one prior study using MRI extends beyond the kidneys to also cover the liver, but not the spleen, using a 2D U-net trained with coronal T2 fat-suppressed images acquired from 2007–2015 from 145 patients. Currently, T2-weighted fast spin echo images are more routinely acquired in the axial plane and generally without fat saturation when using the single shot technique.

Herein, an open-source deep learning method for segmenting kidneys on axial T2-weighted abdominal MRI as deployed in Goel et al. [22] is extended to include liver and spleen segmentation automatically for a more comprehensive assessment of ADPKD. Segmentation accuracy was evaluated with both external and prospective validations using radiologist-corrected contours as the standard of reference. Interobserver reproducibility and time required for model assisted organ volume measurements were compared to manual contouring on a subset of prospective cases.

## 2. Materials and Methods

### 2.1. Patients

This HIPAA compliant study was approved by the Institutional Review Board of Weill Cornell Medicine. Subjects (n = 215) enrolled in the Rogosin Polycystic Kidney Disease Data Repository signed informed consent and their MRIs were used for algorithm development and training. Thirty additional subjects with ADPKD who had abdominal MRI performed at outside institutions, but whose images were stored in Weill Cornell Picture Archiving and Communication System (PACS), were used for external validation. An additional 30 subjects who were consecutively imaged after deployment of the algorithm into the MRI workflow were used for prospective evaluation. The retrospective review of these images and clinical data was approved by the Weill Cornell Institutional Review Board (IRB); the requirement for informed consent was waived. Clinical information from these subjects was obtained from the Rogosin PKD Repository, including serum creatinine, height, weight, age, gender, and race. Estimated glomerular filtration rate (eGFR) was determined based upon the CKD-EPI method [36]. Mayo classification of ADPKD subjects was determined using https://www.mayo.edu/research/documents/pkd-center-adpkd-classification/doc-20094754 (accessed on 5 April 2022). All images, clinical and laboratory data were coded to maintain subject confidentiality.

### 2.2. MR Imaging

Imaging data used for training were acquired with the routine clinical protocol without any special preparation at 1.5T or 3T using body array coils on multiple MRI scanners (Architect, Artist, MR450, MR750, HDXT, GE Healthcare, Waukesha WI and Aera, Skyra, Vita, Siemens Healthineers, Erlangen, Germany). Pulse sequences acquired on the patients included axial T2, coronal T2, axial 3D spoiled gradient echo with either fat suppression or Dixon fat water separation, axial steady state free precession (SSFP), coronal SSFP and axial diffusion weighted imaging from mid-chest to below the bottom edge of kidneys. Because the axial T2 weighted images provided the best contrast between the organs and the background, they were routinely used at our institution for deriving organ volumes by manual contouring and have been used in prior deep learning studies of kidney segmentation [22]; this sequence was selected for the multiorgan segmentation. Axial T2 DICOM tag details are provided in Table S1.

### 2.3. Labeling Training Data

All labeling was performed on ITK-SNAP (version 3.8.0, Paul A. Yushkevich, Philadelphia, PI, USA) by four observers (AG, HD, SR, AS) with at least one year of labeling experience with all labeling reviewed and further refined by a board-certified radiologist (MRP) with >25 years’ experience. For 260 axial T2 weighted scans from 215 patients, the right kidney was labelled 1 (red), the left kidney was labelled 2 (green), the liver was labelled 4 (yellow), and the spleen was labelled 3 (blue). Since model performance on the kidneys was better without distinguishing right from left [22], the green left kidney label was changed to match the red left kidney label prior to model training.

### 2.4. Data Preparation

Axial T2 weighted DICOM images were converted into the NIfTI (Neuroimaging Informatics Technology Initiative) file format using the NiBabel package [37]. Min-max normalization was applied with the value of minimum pixel on the image transformed to 0.0 and the maximum value transformed to 1.0. Each image was mapped to 640 × 640 voxels and cropped down to 512 × 512 to focus on the anatomy of interest. The data was then augmented using affine and computer vision based augmentation transforms using the Python albumentations library [38].

### 2.5. Stratification

Images from subjects who were scanned more than once were grouped together so that stratifications could be performed on a per-subject basis. ADPKD subjects were split into a training data set (n = 151 subjects, 6392 images prior to albumentation) and a validation data set (n = 64 subjects, 3148 images) stratified by TKV and pulse sequence name (an indicator of the scanner utilized). Cross validation was not practical on our computer due to time constraints. A hold out test set was not necessary because both external and prospective validations were performed.

### 2.6. Deep Learning Model Training

**Figure 1:**
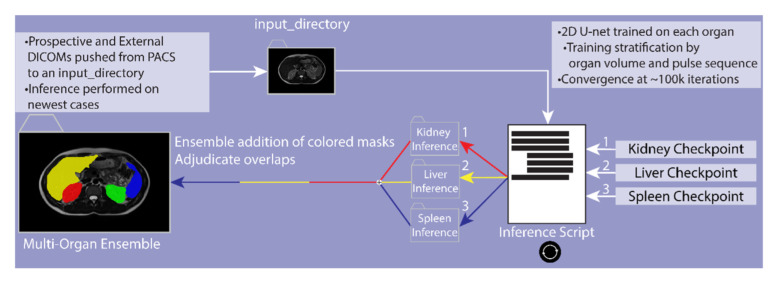
DICOMs of prospective and external cases are pushed to a root input directory from PACS. Checkpoints of each organ are then used to perform organ-specific inference. Once all inferences are complete, the algorithm combines the organs and adjudicates the model overlaps to create a multi-organ ensemble.

Training was performed on a workstation with 4 GeForce GTX TITAN X 12GB GDDR5 GPUs. Our 2D model utilized a CNN architecture based on a semantic segmentation approach written in Python, with Pytorch segmentation libraries [22]. A U-net model was trained over a pretrained EfficientNet encoder using soft dice loss and cross entropy loss. For model encoder, efficient net was used within the Pytorch segmentation libraries [39]. The model decoder was composed of five up sampling layers after using the default SIMP library [39]. After each epoch, validation was performed, saving the best model checkpoints until convergence (Figure 1). Ongoing validations with each epoch visualized in TensorBoard were used to determine convergence.

### 2.7. Resolving Conflicts between Different Organs

**Figure 2:**
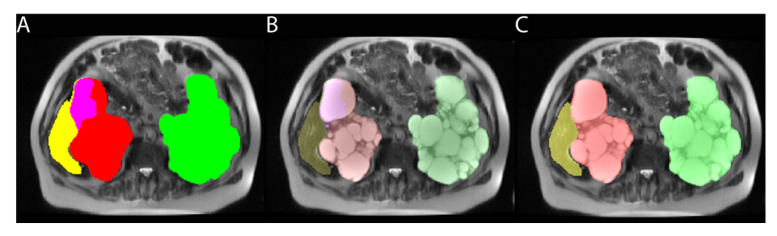
Axial T2 weighted image of a 67-year-old male with ADPKD showing: (**A**) pink voxels between liver (yellow) and right kidney (red) because they met the 2D model criteria for both kidney and liver. (**B**) same image as (**A**) but with increased transparency of labels to show that the pink overlap voxels correspond to a right renal cyst. (**C**) corrected image with overlap voxels now assigned to red corresponding to the right kidney.

The model output for each organ was a probability map indicating the likelihood that each voxel was from that organ. A threshold 50% probability was used as the cutoff to define positive voxels for each organ. Conflicts arose when a voxel was above the 50% probability threshold for more than one organ. For example, voxels on the border between the kidney and liver might be above the 50% threshold for both kidney and liver. Initially, these conflicts were resolved by assigning a new color defined by the sum of the two colors, which is automatically allocated in applications such as ITK-SNAP, Figure 2. For example, in the case of right kidney and liver overlap, the right kidney (red = 1) added with the liver (yellow = 4) was assigned to pink (5). This choice was implemented to allow the radiologist to adjudicate the overlap. However, single voxels of an overlap color could be difficult to find and cumbersome to replace with their correct color, based upon expert visual inspection. With experience adjudicating overlap voxels, it was apparent that most kidney/liver overlapping voxels were assigned to the kidney. Consequently, the algorithm was adjusted to assign right kidney/liver overlaps to default to the right kidney mask. Likewise, overlapping voxels between the left kidney, spleen and liver were satisfactorily resolved by assigning first choice to the left kidney, then the spleen and, finally, the liver. Since right/left kidneys were grouped together to improve model performance, both kidneys were labeled red on the model output. This was corrected by assigning green to all kidney voxels left of the midline.

### 2.8. Image Cropping

**Figure 3:**
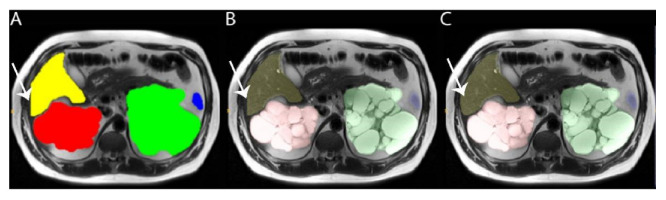
Axial T2 weighted image from a 59-year-old male with ADPKD showing: (**A**) model correctly labeling right kidney (red), left kidney (green), spleen (blue) and liver (yellow) except that inference cropping causes an incorrectly straight liver label border (white arrow). (**B**) with partial label transparency, the underlying organs are visualized with numerous cysts in both right and left kidneys (red and green), including the incorrectly straight liver label border (white arrow). (**C**) after removing the cropping step from the inference input, the liver border is correctly labeled without cropping (white arrow).

For a few cases in which massively enlarged organs were close to the image boundary (within 64 voxels), there were cropping artifacts. Figure 3 illustrates an example of cropping artifact observed for liver segmentation utilizing the same cropping optimized for kidneys. Training the model without cropping, however, was associated with decreased model performance. After experimenting with using less cropping, a robust outcome with excellent model performance over a wide spectrum of cases was achieved using standard symmetrical cropping during training but without expansion and cropping on validation. This may have decreased model performance slightly but was overall better for model assisted annotations because it eliminated the need to manually correct cropping artifacts. Replacing the validation expansion and cropping with generalized resizing also eliminated the need for complex inference transformations.

### 2.9. External and Prospective Validation

Abdominal MRI (n = 30) from outside hospitals on ADPKD subjects, stored in the Weill Cornell PACS, including axial T2 weighted images, were used for external validation. After running the model inference, the contours were corrected using ITK-SNAP by an expert observer (AS) and checked by a board-certified radiologist (MRP) with over 20 years of experience measuring kidney volumes on MRI. The corrected contours were utilized as the gold standard truth for calculating root mean squared error, mean percent error and DICE scores.

After the training was complete and the model inference became operational, this model was implemented into the routine analysis of research registry patients using the same GPU server utilized for training, which was located within the PACS firewall to maintain cybersecurity. As subjects were scanned, their axial T2 weighted images were pushed to the GPU server to run the model inference. After running the inference, the contours were manually corrected by a board-certified radiologist (MRP) as necessary and were saved on the deep learning server within the PACS firewall for future reference, review, display at clinical conferences and sharing with referring physicians. Organ volumes calculated from manually corrected model segmentations were included in the MRI reports. The corrected contours were considered to be ground truth to calculate the Dice score. The first consecutive 30 ADPKD subjects imaged by abdominal MRI were utilized for this prospective validation.

### 2.10. Time Savings and Reproducibility with Model Assisted Contouring

For the first 10 prospective cases, three expert observers (AS, HD, SR), with experience contouring more than 50 cases each both contoured manually and with model assistance to assess the potential time savings and improvement in inter-observer agreement. Manual and model assisted contouring was performed in random order with one week in between to eliminate the potential for confounding effects of operator memory of the organ boundaries. Reproducibility among the three observers was assessed for the manual and model assisted contouring to determine if the model improved reproducibility.

### 2.11. Statistical Analysis

Continuous variables were reported as mean (±SD) or median (inter quartile range [IQR]) in case of violation of normal distribution. Categorical variables were reported as frequency (%). Manual contouring (ground truth) was compared to automated segmentations by calculating the Dice similarity coefficient (DSC) between mask sets A and B as

(1) $$\mathrm{DSC}=\frac{2\left|A\cap B\right|}{\left|A\right|+\left|B\right|}$$

where ∩ is the intersection operator, A is the ground truth, B is the model output, and |S| is the cardinality, or size, of a set. From the definition, DSC ranges from 0 to 1; perfect agreement results in DSC = 1.0 [40]. Root mean square error, average percent error and concordance coefficient [41] were also calculated on external and prospective test sets between the ground truth and model output. Reproducibility among the three observers for manual contouring and model assisted contouring was assessed by calculating the standard deviation of the values of the three observers. A Bland-Altman analysis was performed to evaluate for the percent error of TKV between the reference standard and the automated method [42].

## 3. Results

Demographic details of the 215 patients used for training/validation as well as the 30 ADPKD external validation and 30 consecutive ADPKD prospective validation cases were similar, as shown in Table 2. For the training data, ADPKD subjects were 54% female and 46% male. The mean GFR was 68 mL/min (Table 2). The distribution of Mayo Classifications [10] was A-13%, B-27%, C-33%, D-16% and E-11%.

### 3.1. Model Accuracy

A 2D U-net model was trained with an EfficientNet as its backbone pretrained on the ImageNet. During the training, the rectified Adam (RAdam) and the Lookahead optimizers were utilized to optimize the convergence. With a batch size of 8, convergence was achieved over about 100 epochs for training on each organ. Initial analysis of model performance on training data showed DSC = 0.94 for the left kidney, 0.93 for the right kidney, 0.94 for the liver and 0.91 for the spleen, indicating no issue with underfitting. There was also no evidence of overfitting with favorable DSC, concordance coefficients, root mean square error and average percent error. The number of cases with zero error for kidneys, liver and spleen are shown in Table 3 for (A) external validation and (B) prospective validation. Notably, the performance on external validation is similar to the performance on prospective cases, suggesting that there is good generalizability of model performance beyond the training cases. These kidney segmentation results with DSC of 0.95 to 0.98 are superior to most prior studies which range from 0.80 to 0.98, and these kidney results are comparable to Goel et al. [22], on which this work is based, see Table 1. For liver segmentations the 0.96 to 0.97 DSC reported here is slightly better than the DSC of 0.95–0.96 reported in the only MRI paper analyzing liver in addition to kidneys (van Gastel et al.) [32]. The especially impressive spleen results where DSC = 0.98–0.99 had not been previously reported for ADPKD.

**Figure 4:**
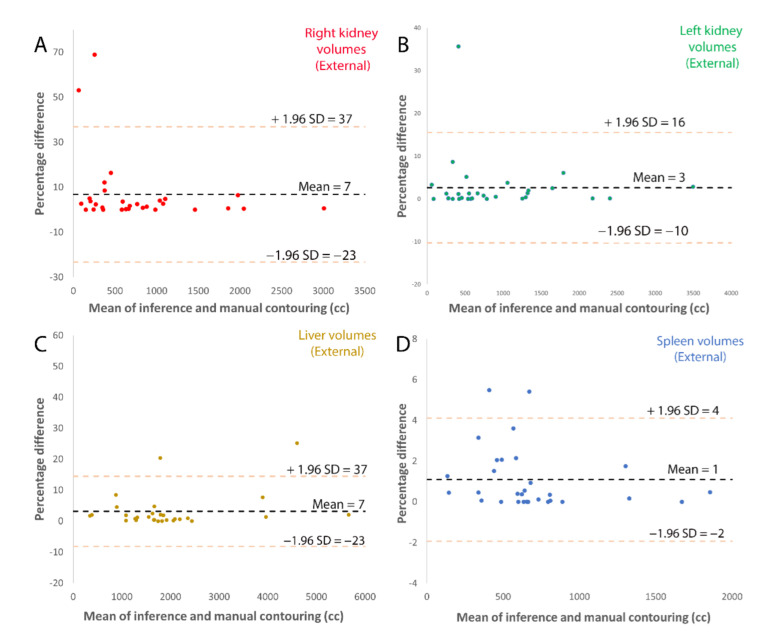
Bland-Altman plots of external validation comparing the inference volume with radiologist corrected volume for (**A**) left kidney, (**B**) right kidney, (**C**) spleen and (**D**) liver.

**Figure 5:**
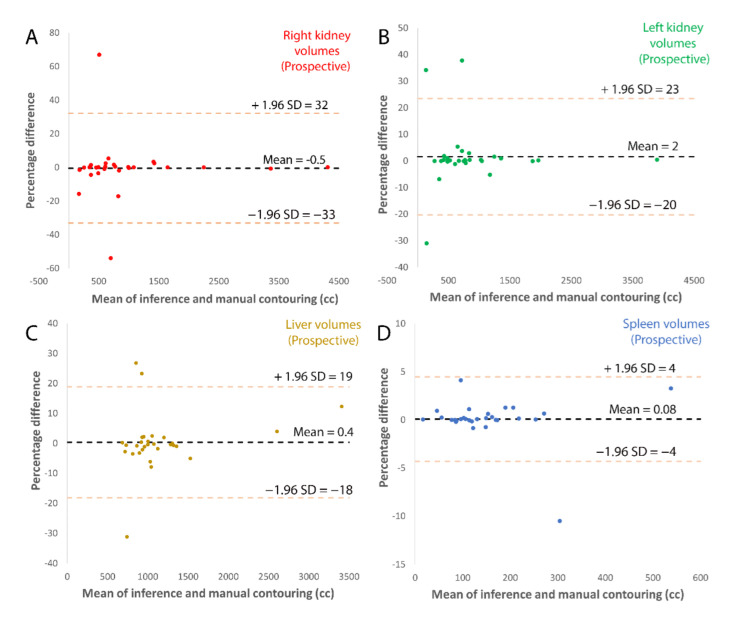
Bland-Altman plots of prospective validation comparing the inference volume with radiologist corrected volume for (**A**) left kidney, (**B**) right kidney, (**C**) spleen and (**D**) liver.

**Figure 6:**
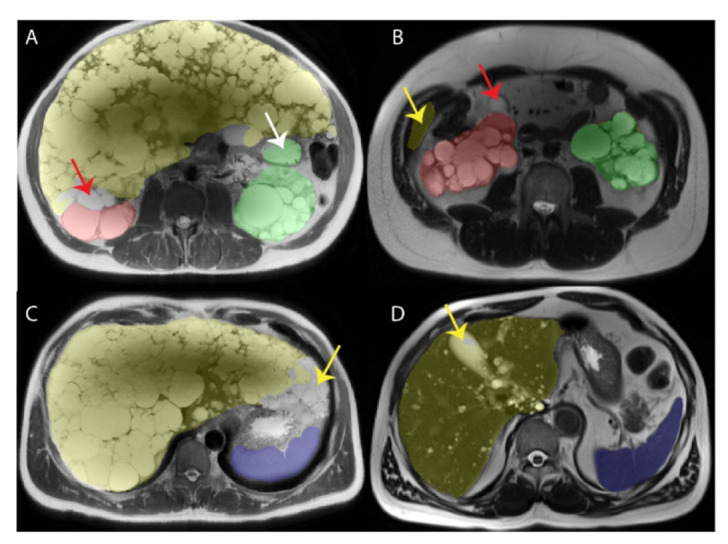
Examples of model error on axial T2. (**A**) Axial T2 weighted image from a 51-year-old male with ADPKD showing an unlabeled cystic region (red arrow) between liver (yellow) and right kidney (red) reflecting the challenge of finding the boundary between the right kidney from the liver, with both being very cystic. Also note that the fluid filled stomach (white arrow) is incorrectly labelled as left kidney (green). (**B**) Axial T2 weighted image from a 59-year-old male with ADPKD demonstrating correct labeling of the left kidney (green), incorrectly labelled abdominal wall mistaken for liver (yellow arrow), and an unlabeled anterior right renal cyst (red arrow) which should be red for right kidney but is in a location which can be mistaken as gallbladder. (**C**) Axial T2 weighted image from a 51-year-old male with ADPKD demonstrating (**A**) correct labeling of the spleen (blue) but incomplete labeling (yellow arrow) of the left edge of a massively enlarged liver which does not commonly extend this far to the left side of the patient. (**D**) Axial T2 weighted image from a 62-year-old male with ADPKD showing near perfect labeling of the spleen (blue). The gallbladder (yellow arrow) is partially incorrectly labeled as liver (yellow).

Bland-Altman plots for the external validation, Figure 4, and prospective validation, Figure 5, show that most ADPKD subjects had excellent model performance that was better than the Table 3 average values. However, a few outlier cases, where the model performed poorly, had large errors. These outliers tended to have larger volumes compared to ground truth, which created bias that was prominent at the lower organ volumes, especially for kidneys. This may be a limitation of this methodology for patients with smaller TKV. Examples of model performance and typical errors are illustrated in Figure 6.

### 3.2. Time Savings with Model-Assisted Annotation

The mean times for manual segmentations and model assisted segmentations for liver, kidneys and spleen are shown in Table 4. Overall, using the model reduced the radiologist time required for segmentations by 42% from 33:04 to 19:17 min (*p* = 0.001).

### 3.3. Interobserver Variability Improvement with Model Assisted Annotations

The mean organ segmentation values were nearly identical for the manual and model assisted annotation groups, indicating that both methods were on average producing similar results. To determine if interobserver variability of the segmentation was reduced with model assisted segmentations, standard deviations were calculated, (see Table 5), as well as interclass correlation coefficients among the three observers for the manual and model assisted segmentations. The smaller mean standard deviations and higher ICCs = 1.000, 1.000, 1.000, 0.999 (for right kidney, left kidney, liver and spleen, respectively) for model assisted, compared to manual, 0.999, 0.999, 0.995 and 0.994, respectively indicate an improvement in reproducibility of the measurement (*p* < 0.05) for all organs with model assistance.

**Figure 7:**
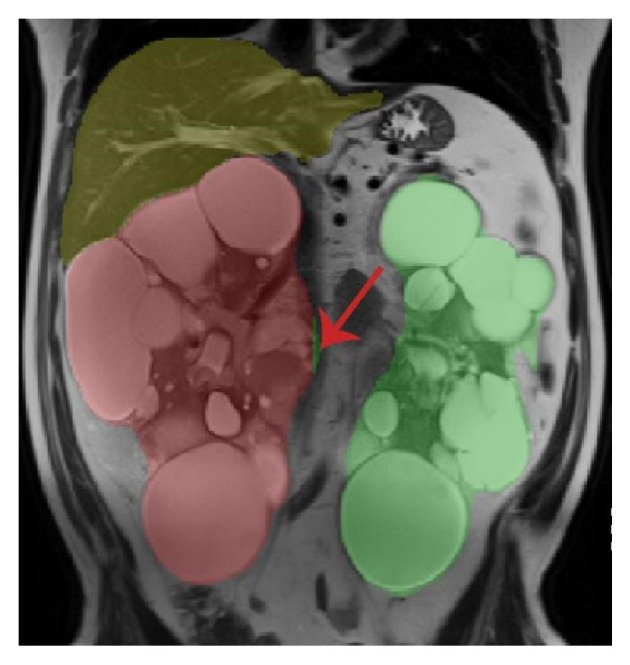
Coronal T2 weighted image from a 61-year-old male with ADPKD showing correctly labeled liver (yellow) and left kidney (green). Right kidney (red) is mostly correct but has a thin vertical sliver (red arrow) that crossed the midline and became labeled as left kidney (green).

Interestingly, even though the algorithm was trained on axial T2 images it also worked well on coronal images (see Figure 7), further supporting the generalization of the model.

## 4. Discussion

These data from 275 ADPKD patients (151 training, 64 internal validation, 30 external validation, 30 prospective validation) and a total of 318 MRI exams show that a simple 2D deep learning U-net model facilitates accurate organ volume measurements and reduces the time for model assisted segmentations. Final ground truth results were similar between manual and model assisted segmentations, with substantially improved interobserver agreement with model assisted annotations. Accordingly, measurements by a single observer are likely to be more accurate and more rapid with model-assisted segmentation. This improvement in reproducibility is especially useful when monitoring slowly progressive diseases, such as ADPKD where organs enlarge by only 5% per year [43], reducing measurement variability from 10% to 5% enables meaningful measurements to be made at annual rather than biennial intervals. This work builds on prior models measuring only kidney [22] or combined kidney and liver [32] for a more comprehensive assessment of ADPKD disease progression.

Radiologists and image post-processing specialists will prefer this model assisted annotation methodology because it allows for gold standard of reference, manual segmentations to be performed in substantially less time. Since the deep learning segmentation is doing the bulk of the work, the radiologist can focus on the fine details when correcting the model output to ensure a perfect segmentation. Indeed, model assisted segmentations by multiple observers in this study had substantially reduced standard deviations, indicating an improvement in measurement reproducibility over manual contouring without model assistance. These corrected studies can be used to further train the model for constant, never-ending improvement in the methodology. This is especially important for MRI where minor improvements in the imaging technology are periodically implemented and need to be incorporated into the model. Unlike prior reports attempting to fully automate organ volume measurements into ADPKD with accuracy approaching manual contouring [22,23,24,25,26,27,28,29,30,31,32,33,34,35], this research demonstrates superior measurement reproducibility over manual contouring that can readily adapt to technological advances. Since the deep learning server is within the PACS firewall, technologists can rapidly transfer images to the server for running the inference. All the annotations are saved in a cyber-secure environment for later review as necessary and for use in further model training. Although the reports contain only the final volume measurements, access is possible for sharing with referring physicians and displaying the annotations at clinical case management conferences.

Most prior deep learning approaches to ADPKD focused only on measuring kidney volumes. The one study using MRI data to measure both kidney and liver volumes was based upon coronal T2 fat suppressed images obtained in the 2007 to 2015 timeframe, which are not currently acquired at many institutions including the Weill Cornell and Columbia University Medical Centers. This study utilizes the more ubiquitous axial T2 weighted single shot fast spin echo type images without requiring fat suppression, and analyzes all abdominal organs enlarged in ADPKD, kidneys, liver and spleen.

Time saving was greatest for the spleen (50%), most likely due to the homogeneous signal intensity of the spleen and the absence of indistinct organ boundaries. The least time saving was for the liver (31%) reflecting the challenge of defining its boundaries abutting the stomach, heart, pancreas, lung, chest wall vasculature (IVC, aorta, portal vein) and especially the right kidney. The slightly greater time saving for the left kidney compared to right kidney likely reflects the relatively high degree of difficulty defining the border between the right kidney and the liver (Figure 6), which is especially difficult when both organs have numerous cysts.

Since each organ was segmented independently with its own binary 2D U-net, adjudicating overlap voxels involving more than one organ was essential. Our initial approach of highlighting overlap voxels for manual adjudication by assigning new colors was cumbersome. Defaulting to the most common overlap corrections became an effective, time efficient solution. Another strategy would be to utilize a multi-class model implementation via softmax adjudication. Our attempts at multiclass modeling were not successfully converging. Similarly, one might expect 3D models to perform better than our simple 2D model. However, 3D models require greater computer memory and more data. If each MRI exam was a single 3D model data point, we would have an N of 260 instead of 9540 individual 2D images before augmentation. Adding additional tissues and organs to the ensemble, as well as subsegmenting organs to measure cyst fraction or cyst volumes, is easier with the 2D method, which does not require every tissue/organ to be labeled on every case used for training

Measuring organ volumes is important in ADPKD for determining prognosis (e.g., age of onset of ESKD). Other potential benefits that might accrue by improving the accuracy and efficiency of TKV measurement includes the assessment of indications for and responses to treatment with tolvaptan to slow progression of CKD and for assessing other therapies [44]. Monitoring liver and spleen volumes may be similarly useful in those ADPKD subjects where those organs are most affected.

The main strength of this study is the large number of ADPKD subjects used for training with contours verified with two trained observers including a board-certified radiologist experienced with the radiographic evaluation of ADPKD patients. A limitation is the predominant enrollment of patients with more advanced cystic disease with high total kidney volumes. Another limitation is that the 2D methodology does not allow the deep learning model to consider 3D features, especially information on adjacent slices, which has been useful during manual contouring. Yet another limitation is the challenge of separating right and left kidneys when they are really large and cross the midline (Figure 7). The excellent performance on training, prospective and external data sets indicates that there is no problem with underfitting or overfitting the training data. However the Bland-Altman analysis shows outliers with large errors for the subjects with smaller TKV, presumably earlier in the disease process. This raises the possibility of reduced accuracy for smaller TKV, and large errors were also seen for small spleens. This algorithm is not considered fully automatic since we are routinely correcting the model output. However, these model-assisted annotations for calculating organ volumes are more reproducible and likely more accurate compared to manual only annotations of the past. Finally, it only works on axial T2 weighted images.

Future work will extend segmentations to more pulse sequences and imaging planes as well as more tissues including peritoneal fat, pancreas, pleural effusions as well as including sub-segmentations of cyst factions, exophytic cysts [17], and simple versus complex cysts [45]. Extending this ensemble methodology to additional organs is facilitated by the 2D approach which does not require training data to have every structure labeled for every patient. However, extending it to 3D may improve performance by incorporating of 3D information into the deep learning model. Plans are also underway to extend it to CT data to determine which modality provides the most accurate and reproducible measurements. Ongoing clinical, model assisted segmentations will continue to expand the database of training cases, likely further improving model performance and potentially obviating the need for model segmentation corrections. The automated segmentation of more pulse sequences may allow for better quality control and further improvements in interobserver variability.

## Figures and Tables

**Table 1:** Literature on deep learning methods for organ volume measurements in ADPKD.

First Author	Modality	Year	ADPKD subjects	Segmentation Methodology	Dice Score	Other Metrics	Organ
Sharma [26]	CT	2017	125	2D VGG-16 FCN	0.86	7.8%	Kidney
Keshwani [24]	CT	2018	203 CT scans **	Multi-task 3D FCN	0.95	3.86%	Kidney
Shin [27]	CT	2020	214	3D V-net	0.961 *	95% within 3%	Kidney + Liver
Onthoni [25]	CT	2020	97	2D SSD Inception Network V2	-	Images: mAP: 94% Subjects: mAP: 82%	Kidney
Hsiao [23]	CT	2022	210	FPN + EfficientNet	0.969	-	Kidney
Jagtap [28]	US	2022	22	2D U-Net	0.80	4.12%	Kidney
Kim [29]	MRI Cor T2 fatsat	2016	60	SPPM + PSC	0.88	MCC: 0.97	Kidney
Kline [30]	MRI Cor T2	2017	2000 scans **	2D U-Net + ResNet-like encoder	0.97	0.68%	Kidney
Guangrui [31]	MRI Axial + Cor T1	2019	305	3D VB-Net ***	RK-0.958 LK-0.965	-	Kidney
Van Gastel [32]	MRI Cor T2 fatsat	2019	145	2D U-Net	-	LK: 0.96 RK: 0.95 TKV: 0.96 Liver: 0.95	Kidney + Liver
Kline [33]	MRI Cor T2 +/−fatsat	2020	60	2D U-Net + ResNet-like encoder	1st Reader: 0.86 2nd Reader: 0.84	1st Reader: 3.9% 2nd Reader: 8%	Kidney cysts
Goel [22]	MRI Axial T2	2022	173	2D U-Net + EfficientNet encoder	External: 0.98 Prospective: 0.97	External: 2.6% Prospective: 3.6%	Kidney
Raj [34]	MRI Cor T1	2022	100	2D Attention U-Net	0.922	MSSD: 0.922 and 1.09 mm	Kidney
Taylor [35]	MRI	2022	227 Scans	3D U-Net	0.96 each kidney	LK:1.8% RK:1.79%	Kidney

FPN = Feature Pyramid Network; FCN = Fully Convolutional Network; SSD = Single Shot Detector; MSSD = Mean Symmetric Surface Distance; RK= right kidney; LK = left kidney; Cor = Coronal; MAPE= Mean absolute percentage error; mAP= mean Average Precision; MCC = mean correlation coefficient. * DSC corresponds to combination of TKV and liver volume. ** number of subjects is unknown. *** customization of V-Net.

**Table 2:** Demographic data.

Parameter	Training/Validation Data	External Test Set	Prospective Test Set
Number of Patients	215	30	30
Number of MR exams	260	30	30
DICOM images	9540	1368	2137
Male:Female (%male)	98:117 (46%)	17:13 (57%)	11:19 (37%)
Age at scan (years)	49 ± 14	49 ± 16	46 ± 15
eGFR (mL/min/1.73 m^2^)	68 ± 28	85 ± 30	72 ± 34
Total Kidney Volume (mL) *	1287 (669–2213)	1334 (693–2376)	1444 (885–2020)
Ht-TKV (mL/m) *	757 (415–1275)	777 (393–1297)	837 (550–1234)
*Mayo class* ** -Report N and %			
A	29 (13%)	4 (13%)	1 (3%)
B	58 (27%)	8 (27%)	6 (20%)
C	70 (33%)	7 (24%)	13 (44%)
D	34 (16%)	10 (33%)	7 (23%)
E	24 (11%)	1 (3%)	3 (10%)
*Race*-Report N and %			
Asian	10 (5%)	1 (3%)	4 (13%)
White	148 (69%)	23 (77%)	16 (53%)
Black	14 (6%)	1 (3%)	2 (67%)
Unknown	43 (20%)	5 (17%)	8 (27%)

* median (interquartile range), ** Calculated based upon the first exam for subjects with multiple MRIs. Ht-TKV: height-adjusted total kidney volume.

**Table 3:** (**A**) Model accuracy on external validation (n = 30), median (interquartile range). (**B**) Model accuracy on prospective validation (n = 30), median (IQR).

(A)				
External Test Set	Right Kidney	Left Kidney	Liver	Spleen
Model volume (mL) Corrected model volume (mL)	617 (327–1009) 608 (316–1041)	582 (416–1289) 582 (365–1285)	1706 (1292–2087) 1684 (1287–2076)	220 (145–274) 222 (157–280)
DSC	0.96	0.98	0.97	0.96
Concordance Coefficient	>0.99	>0.99	0.98	0.99
RMS error (mL)	42	39	258	17
Average % error	7%	3%	3%	1%
Number with zero error	5 (17%)	6 (20%)	1 (3%)	7 (23%)
**(B)**				
**Prospective Test Set**	**Right Kidney**	**Left Kidney**	**Liver**	**Spleen**
Model volume (mL) Corrected model volume (mL)	625 (370–1000) 650 (394–998)	729 (481–1039) 768 (485–1043)	1711 (1489–2065) 1727 (1495–2051)	244 (177–315) 241 (175–318)
DSC	0.96	0.96	0.96	0.95
Concordance Coefficient	>0.99	>0.99	>0.99	0.98
RMS error (mL)	112	65	112	37
Average % error	6%	5%	5%	1%
# with zero error	2 (7%)	3 (20%)	2 (7%)	2 (7%)

**Table 4:** Mean time (minutes) for manual organ segmentation and model assisted segmentations for the first 10 prospective cases averaged over four trained observers.

	Manual Segmentation	Model Assisted Segmentation	Time Savings	*p*-Value
Right Kidney	7:39 ± 2:26	4:31 ± 1:34	3:08 (41%)	0.004
Left Kidney	7:34 ± 3:44	4:16 ± 1:35	3:19 (44%)	0.01
Liver	12:49 ± 6:10	8:49 ± 3:52	3:59 (31%)	0.007
Spleen	4:13 ± 0:48	2:04 ± 0:59	2:09 (51%)	0.0003
Total	33:04 ± 8:05	19:17 ± 7:19	13:47 (42%)	0.001

**Table 5:** Standard deviations of organ volumes measured by three trained observers for segmentations performed manually or with model assistance.

	Volume Measurement Standard Deviations		
	Manual Segmentation (mL)	Model Assisted Segmentation (mL)	*p*-Value
Right Kidney Left Kidney	14 10	7 5	0.02 0.07
Liver	55	11	0.001
Spleen	14	5	0.001

## Data Availability

Data generated or analyzed during this study as well as the model code and checkpoints are available from the corresponding author by request subject to institutional review and a data use agreement.

## Supplementary Materials

The following supporting information can be downloaded at: https://www.mdpi.com/article/10.3390/tomography8040152/s1, Table S1: Single Shot Fast Spin Echo axial T2-Weighted image DICOM tag details.

## Table of Abbreviations

ADPKD	Autosomal Dominant Polycystic Kidney Disease
CKD	Chronic Kidney Disease
CKD-EPI	Chronic Kidney Disease Epidemiology Collaboration
CNN	Convolutional Neural Network
CT	Computerized Tomography
DICOM	Digital Imaging and Communications in Medicine
DSC	Dice Similarity Coefficient
eGFR	Estimated Glomerular Filtration Rate (based on CKD-EPI method)
ESKD	End Stage Kidney Disease
HASTE	Half Fourier Single-shot Turbo Spin-Echo
MRI	Magnetic Resonance Imaging
NIfTI	Neuroimaging Informatics Technology Initiative
PACS	Picture Archiving and Communication System
SSFP	Steady State Free Precession
SSFSE	Single-Shot Fast Spin Echo
TE	Time to Echo
TKV	Total Kidney Volume
ht-TKV	Height Adjusted Total Kidney Volume
TR	Time to Repeat
