# Supplementary material for: Deep Learning Automation of Kidney, Liver, and Spleen Segmentation for Organ Volume Measurements in Autosomal Dominant Polycystic Kidney Disease
Source: Tomography. 2022 Jul 13;8(4):1804–19. doi: 10.3390/tomography8040152 (PMC9326744; doi:10.3390/tomography8040152)
Supplement: Supplementary file 1 [file tomography-08-00152-s001.zip › tomography-1758429-supplementary.pdf]

Table S1. Single Shot Fast Spin Echo axial T2-weighted image DICOM tag details.

DICOM Tag	Value
Echo Time (msec)	89–185
Slice Thickness (mm)	6–8
Slice Space (mm)	6–8
Matrix (Phase x Frequency)	192-256 x 256
Acquisition Duration (sec)	46–83
